# Comparative Analysis of Complicated Urinary Tract Infections Caused by Extensively Drug-Resistant *Pseudomonas aeruginosa* and Extended-Spectrum β-Lactamase-Producing *Klebsiella pneumoniae*

**DOI:** 10.3390/antibiotics11111511

**Published:** 2022-10-29

**Authors:** Elena Sendra, Inmaculada López Montesinos, Alicia Rodriguez-Alarcón, Juan Du, Ana Siverio-Parés, Mar Arenas-Miras, Esperanza Cañas-Ruano, Nuria Prim, Xavier Durán-Jordà, Fabiola Blasco-Hernando, Enric García-Alzorriz, Francesc Cots, Olivia Ferrández, Silvia Gómez-Zorrilla

**Affiliations:** 1 Infectious Diseases Service, Hospital del Mar, Infectious Pathology and Antimicrobials Research Group (IPAR), Institut Hospital del Mar d’Investigacions Mèdiques (IMIM), Universitat Autònoma de Barcelona (UAB), Universitat Pompeu Fabra (UPF), 08003 Barcelona, Spain; 2 Pharmacy Service, Hospital del Mar, Infectious Pathology and Antimicrobials Research Group (IPAR), Institut Hospital del Mar d’Investigacions Mèdiques (IMIM), Universitat Autònoma de Barcelona (UAB), Universitat Pompeu Fabra (UPF), 08003 Barcelona, Spain; 3 Microbology Service, Laboratori de Referència de Catalunya, Hospital del Mar, 08003 Barcelona, Spain; 4 Methodology and Biostatistics Support Unit, Institut Hospital del Mar d’Investigacions Mèdiques (IMIM), 08003 Barcelona, Spain; 5 Management Control Department, Hospital del Mar-Parc de Salut Mar, 08003 Barcelona, Spain; 6 Spanish Network for Research in Infectious Diseases (REIPI), Center for Biomedical Research in Infectious Diseases Network (CIBERINFEC), Instituto de Salud Carlos III, 28029 Madrid, Spain

**Keywords:** *Pseudomonas aeruginosa*, extensively drug-resistant, *Klebsiella pneumoniae*, extended-spectrum beta-lactamase-producing, multidrug resistance (MDR), urinary tract infections

## Abstract

The objective was to compare clinical characteristics, outcomes, and economic differences in complicated urinary tract infections (cUTI) caused by extensively drug-resistant *Pseudomonas aeruginosa* (XDR *P. aeruginosa*) and extended-spectrum beta-lactamase-producing *Klebsiella pneumoniae* (ESBL-K. *pneumoniae*). A retrospective study was conducted at a tertiary care hospital. Patients with XDR *P. aeruginosa* and ESBL-*K. pneumoniae* cUTIs were compared. The primary outcome was clinical failure at day 7 and at the end of treatment (EOT). Secondary outcomes: 30- and 90-day mortality, microbiological eradication, and economic cost. Two-hundred and one episodes were included, of which 24.8% were bloodstream infections. Patients with XDR *P. aeruginosa* cUTI more frequently received inappropriate empirical therapy (*p* < 0.001). Nephrotoxicity due to antibiotics was only observed in the XDR *P. aeruginosa* group (26.7%). ESBL-*K. pneumoniae* cUTI was associated with worse eradication rates, higher recurrence, and higher infection-related readmission. In multivariate analysis, XDR *P. aeruginosa* was independently associated with clinical failure on day 7 of treatment (OR 4.34, 95% CI 1.71–11.04) but not at EOT, or with mortality. Regarding hospital resource consumption, no significant differences were observed between groups. XDR *P. aeruginosa* cUTI was associated with worse early clinical cures and more antibiotic side effects than ESBL-*K. pneumoniae* infections. However, no differences in mortality or in hospitalization costs were observed.

## 1. Introduction

*Pseudomonas aeruginosa* has an extraordinary ability to develop resistance through chromosomal mutations and the acquisition of resistance genes [1,2,3]. The emergence of infections caused by multidrug-resistant and extensively drug-resistant *Pseudomonas aeruginosa* (MDR *P. aeruginosa* and XDR *P. aeruginosa*) has become a serious global concern [4]. Indeed, *P. aeruginosa* is considered a difficult-to-treat bacteria and is included among the ESKAPE pathogens [5].

*Klebsiella pneumoniae*, another of the ESKAPE microorganisms, is one of the six major pathogens responsible for deaths associated with resistance [6]. Healthcare-related infections caused by extended-spectrum beta-lactamase (ESBL)-producing *Klebsiella* spp have increased dramatically over the last few years [7]. The WHO lists both XDR *P. aeruginosa* and ESBL-producing *Klebsiella pneumoniae* (ESBL-*K. pneumoniae*) as of critical priority on their list of pathogens for which new antibiotics are urgently needed [8]. 

Urinary tract infections (UTIs) are the result of the interaction between host susceptibility and bacterial virulence. Host factors include age, gender, and functional or structural abnormalities of the urinary tract [9]. Bacterial virulence factors, predominantly located on cell surfaces, include fimbriae with adhesive tips, protectins, bacterial capsule and lipopolysaccharide (LPS), production of toxins such as haemolysin, and a colony necrotising factor [10]. In terms of age and sex distribution, women have a higher incidence of UTIs than men in every age category, with an estimated life time prevalence of 50% in women and 13.7% in men [11]. According to the Global Burden of Disease Study, the number of incident cases in 2019 increased with age, with a peak incidence in the 30–34 age group for females and >80 years for males [12]. UTIs represent one of the most common healthcare-associated infections, with an estimated prevalence of 12.9% in the United States, 19.6% in Europe, and up to 24% in developing countries [13]. High rates of MDR have been observed in recent years in healthcare-associated UTIs [14,15], which implies very limited treatment options and consequently worse outcomes [16].

While XDR *P. aeruginosa* infection is associated with hospital settings in patients with pre-existing diseases and predisposing factors such as immunodepression [17], ESBL- Enterobacterales infection affects a less immunocompromised population and may be community-acquired [18,19]. Regarding Enterobacterales, although ESBL-producing *Escherichia coli* is more prevalent than ESBL-producing *K. pneumoniae,* we selected the latter microorganism because of its significant clinical impact both as a nosocomial pathogen and in terms of resource consumption.

Previous studies have shown that MDR *P. aeruginosa* is associated with worse clinical outcomes [20,21] as well as a longer hospital stay [22] and increased hospital costs [23]. UTIs caused by ESBL-producers (ESBL-*K. pneumoniae)* versus non-ESBL-producers have also been associated with worse clinical outcomes, including worse clinical cure, higher probability of receiving inadequate empirical treatment, longer hospitalization, higher economic burden, and higher mortality [24,25]. Few studies however have set out to compare whether there are differences between them [26].

The present study aimed to compare the clinical outcome and economic impact of complicated UTI (cUTI) caused by both MDR/XDR Gram-negative bacilli. We hypothesised that cUTI caused by XDR *P. aeruginosa* would be associated with worse clinical (mortality, clinical failure, adverse effects) and economic (higher overall costs) outcomes than those caused by ESBL-*K. pneumoniae*, due to their particularities and difficulties in treatment. [25]. We tested the hypothesis by retrospectively reviewing and including all consecutive adult patients with XDR-*P. aeruginosa* or ESBL*-K. pneumoniae* cUTI at the Hospital del Mar from January 2010 to June 2019 who met the inclusion criteria.

## 2. Methods

### 2.1. Hospital Setting, Study Design, and Study Population

This was a retrospective study, conducted from January 2010 to June 2019 at the Hospital del Mar, a 450-bed tertiary care teaching hospital in Barcelona (Spain). All consecutive adult patients with XDR *P. aeruginosa* or ESBL-*K. pneumoniae* diagnosed with cUTI were retrospectively reviewed. The inclusion criteria were as follows: patients aged 18 years or older with acute pyelonephritis or complicated UTI. Polymicrobial urine culture, non-complicated UTIs, and asymptomatic bacteriuria were excluded. Patients who died in the first 48 h of the episode or were lost to follow-up were also excluded. This paper was written following the STROBE guidelines for observational studies. 

UTI episodes caused by XDR *P. aeruginosa* from January 2010 to June 2019 were included in the XDR *P. aeruginosa* cohort. This cohort was previously analyzed to evaluate the efficacy and safety of aminoglycosides or polymyxin monotherapy compared with other antibiotic regimens [27]. XDR *P. aeruginosa* UTI episodes were matched 1:1 with cases of ESBL-*K. pneumoniae.* UTI ESBL-*K. pneumoniae* episodes were consecutively included from February 2013 until the required sample size was reached November 2019. In total, 201 patients were included: 101 patients in the XDR *P. aeruginosa* cohort and 100 in the ESBL-*K. pneumoniae* cohort. Patients were followed for up to 90 days from the date of the urine culture. In cases of more than one episode of XDR *P. aeruginosa* or ESBL-*K. pneumoniae* UTI, the second and following episodes were assessed if they occurred at least 90 days after the prior one.

### 2.2. Clinical Variables, Data Sources, and Definitions

Three authors (E.S., I.L.M. and S.G.-Z.) were responsible for collecting all data from the hospital’s electronic medical records. Clinical and demographic data were recorded, including age, sex, underlying conditions such as diabetes mellitus, chronic obstructive pulmonary disease (COPD), congestive heart failure, cirrhosis, neurological disorder, hematologic and solid tumor malignancy, as well as the Charlson comorbidity severity index [28]. Finally, the existence of a nephro-urological history was also recorded, including chronic kidney disease, dialysis, renal transplant, urological neoplasia, prior history of benign prostatic hypertrophy, obstructive urinary disease, recurrent UTI, and indwelling urinary catheter or other urological devices in the preceeding 14 days.

With respect to UTI classification, acute pyelonephritis was considered if the patient had at least two of the following criteria: a temperature above 37.7 °C, UTI symptoms (dysuria, urgency, suprapubic pain, and/or pollakiuria), local pain (lumbar back pain and/or pelvic or perineal pain), and/or altered mental status in persons up to 70 years of age. Complicated UTI was established in those patients with the same criteria and a prior history of benign prostatic hyperplasia, intermittent or permanent indwelling urinary catheter (or removal within 48–72 h before the onset of infection), or underlying urological abnormalities such as nephrolithiasis, stricture, stents, history of renal transplantation, urinary diversion or neurogenic bladder.

Acquisition of infection was classified in accordance with Friedman et al. [29] as community-acquired, healthcare-related, or nosocomial. Healthcare-associated risk factors, such as antibiotic exposure, hospital stay, surgery, or ICU admission in the previous three months, and/or residence in a long-term care facility, were also collected. 

Baseline disease severity was measured by the Sequential Organ Failure Assessment (SOFA) score [30], the Quick SOFA score (qSOFA) [31], the Simplified Acute Physiology Score (SAPS II) [32], the need for intensive care unit (ICU) admission and the presence of sepsis or septic shock [31]. In the case of bacteraemia, the Pitt score was also calculated [32].

Recurrence was defined as repeated signs or symptoms of UTI and a urinary isolate with the same susceptibility profile as the index infection. Reinfection was defined as recurrent signs or symptoms of UTI caused by a different strain than the index infection strain. Microbiological eradication was considered if there was no growth of XDR *P. aeruginosa* or ESBL-producing *K. pneumoniae* in the final urine culture, if available. Episodes with missing urine samples during follow-up were classified as indeterminate.

In terms of management, appropriate empirical and definitive antibiotic treatments were assessed. Appropriate antibiotic therapy was considered when at least one antibiotic administered displayed documented in vitro susceptibility in accordance with the breakpoints established by the European Committee on Antimicrobial Susceptibility Testing (EUCAST) [33,34]. Adequate source control was also collected, defined as removal or insertion of an indwelling urinary catheter, percutaneous drainage of the urinary tract (double J stent, nephrostomy), or surgical intervention, as appropriate.

### 2.3. Outcomes and Follow-Up

The primary outcome was clinical failure, determined at day 7 and at the end of treatment (EOT). Clinical failure was considered if there was persistence or worsening of signs and/or symptoms of UTI, modification of the antibiotic therapy due to side effects, and/or death.

Secondary outcomes were 30-day and 90-day mortality, recurrence, microbiological eradication, percentage of infection-related readmissions, and economic cost per episode. Finally, antibiotic-induced side effects were also evaluated. The follow-up period was 90 days from the date of the first urine culture.

### 2.4. Microbiological Studies

Urine cultures were performed as part of the clinical routine, following standard laboratory procedures. Cultures with growth yielding ≥10^5^ colony-forming units/mL of a single bacterial type in a urine sample (collected midstream) were considered positive. Bacterial identification was performed by conventional biochemical tests and matrix-assisted laser-desorption and ionization time-of-flight mass spectrometry (MALDI-TOF MS), using a Bruker Microflex^®^ LT instrument and the MALDI Biotyper^®^ software (Bruker Daltonics, MA, USA). Antibiotic susceptibility testing (AST) of isolates was performed by broth microdilution, using MicroScan panels [Beckman-Coulter] on the automated MicroScan WalkAway system [Beckman-Coulter]. Results were interpreted according to the European Committee for Antimicrobial Susceptibility Testing (EUCAST) guidelines. The following antimicrobials were tested in both species: ceftazidime, cefepime, piperacillin-tazobactam, imipenem, meropenem, aztreonam, ciprofloxacin, gentamicin, tobramycin, amikacin. Amoxicillin-clavulanic acid, cefuroxime, trimethoprim/sulfamethoxazole, and nitrofurantoin, were also tested in *K. pneumoniae*; colistin was assessed in *P. aeruginosa*. Ceftolozane-tazobactam was not routinely used for a large part of the study; it was tested by the gradient diffusion method (Etest, bioMérieux, Marcy-l’Etoile, France) [35] from 2017 onwards. The ESBL screening was based on the AST results of third-generation cephalosporins (MIC > 1 mg/L for cefotaxime/ceftriaxone and ceftazidime) following EUCAST guidelines [29,30]. ESBL production was further confirmed phenotypically by the MIC reduction of any of these cephalosporins combined with clavulanic acid compared with the MIC of the same cephalosporin alone in the microdilution, or by the double-disk diffusion test (DDST) [29,30]. XDR phenotype in *P. aeruginosa* was defined according to Magiorakos et al. [36].

### 2.5. Statistical Analysis

The sample size required (101 per group) was determined from the results of previous studies [19,23] to detect a difference of almost 20% in the 7-day clinical response between XDR *P. aeruginosa* or *ESBL-K. pneumoniae* UTI; statistical power was set at 80%, alpha error at 0.05, and the estimated loss to follow-up at 25%.

Categorical variables were presented as numbers and percentages and were compared by the X^2^ test or Fisher’s exact test. Continuous variables were expressed as the median and interquartile range (IQR) and compared by Student’s t-test or the Mann–Whitney U test, as appropriate. Stepwise logistic regression with variable selection was used to examine independent variables associated with clinical failure (both at day 7 of treatment and at end of treatment). Variables with *p* value < 0.20 in the unadjusted analysis, as well as those that were clinically relevant but not statistically significant, were included in the model. Results were expressed as odds ratios (OR) and its 95% confidence interval (95% CI). An adjusted Cox regression model was used to assess 30-day and 90-day mortality. Variables with *p* value < 0.20 in the unadjusted analysis and/or those considered clinically relevant were included in the model. Results were expressed as hazard ratio (HR) and 95% CI. Finally, unadjusted and adjusted analysis of hospitalization costs were performed through median regression models to assess variables associated with significantly higher costs. The choice of median regression, rather than linear regression, was due to the fact that the distribution of costs was highly skewed to the right (and so did not meet the assumption of normality); *p* < 0.05 was considered statistically significant and all analyses were two-tailed. Statistical analyses were carried out with SPSS Statistics 26.0 software.

### 2.6. Cost Estimation

The Municipal Institute of Health uses a hospital cost accounting system based on full-cost allocation to estimate the direct costs of clinical activity. In the present study, cost estimation was based on a full costing method and the criteria of clinical activity-based costing methods to obtain the highest sensitivity in assessing variability in clinical activity.

Allocation was based on the direct assignment of the cost of the following services to the patient: laboratory, pharmacy, radiology, nuclear medicine, pathology, and prosthetics.

The information systems contain comprehensive data on human resources and their activity: storage, admissions planning, ambulatory, and emergency care, operating rooms, diagnostic and complementary tests, and inter-hospital consultation.

The main economic outcome was the total hospital cost of XDR *P. aeruginosa* and ESBL-*K. pneumoniae* episodes, including fixed costs, variable costs, and pharmacy costs. For the estimation of fixed costs, only episodes related to infection were compared. Fixed costs were derived from surgical procedures, hospitalization, and ICU-stay, and were allocated according to routine criteria: operation or intervention time or number of days of stay in the various hospital units. Variable costs included costs deriving from laboratory, radiology, pathology, prostheses, tests, and pharmacy. Additionally, costs derived from relapses of the infection were collected, including hospital readmissions.

### 2.7. Ethics

The study was approved by the Clinical Research Ethics Committee of the Parc de Salut Mar, Barcelona (register no. 2020/9321). Due to the observational nature of the study and retrospective analysis, the need for written informed consent was waived.

## 3. Results

In total, 1260 urine cultures positive for XDR *P. aeruginosa* or ESBL-*K. pneumoniae* during the study period were included in the preselection process (465 and 795, respectively). Based on the attempted sample size, 201 episodes that met the inclusion and exclusion criteria were included in the final analysis. Seven patients in the XDR *P. aeruginosa* cohort and eight in the ESBL-*K. pneumoniae* group experienced more than one episode. Two patients had infections caused by both microorganisms in different episodes. A total of 50/201 (24.8%) of the episodes were bloodstream infections. 

The epidemiological and clinical characteristics of patients included in the study are shown in Table 1. Male sex was predominant in both cohorts, but significantly higher in the XDR *P. aeruginosa* group than in the ESBL-*K. pneumoniae* group (80 (79.2%) vs. 65 (65%); *p* = 0.025). No significant differences in age or Charlson comorbidity index were observed between the two groups. XDR *P. aeruginosa* episodes were more common in patients who had previously used urinary devices (*p* < 0.001), had urinary tract abnormalities (*p* < 0.001), or had nosocomial acquisition (*p* = 0.005). Patients with cUTI caused by XDR *p. aeruginosa* more frequently received inappropriate empirical therapy than those caused by ESBL-*K. pneumoniae* (80 (79.2%) vs. 44 (44%); *p* < 0.001) and also had longer delays to initiation of appropriate antibiotic therapy (54 (53.5%) vs. 39 (39%); *p* = 0.04). 

The most frequent inappropriate empirical treatments used in the XDR *P. aeruginosa* cUTI group were carbapenems (24 (30%)), piperacillin/tazobactam (19 (24%)), quinolones (9 (11%)) and amoxicillin/clavulanic acid (5 (6%)); the most frequent inappropriate empirical treatments in ESBL-*K. pneumoniae* cUTI were cephalosporins (15 (34%)), piperacillin/tazobactam (11 (25%)), amoxicillin/clavulanic acid (9 (20%)) and quinolones (4 (9%)). There were no differences between the two groups in terms of adequate source control.

Regarding antibiotic susceptibility, in the XDR *P. aeruginosa* group the antimicrobial agents with the lowest resistance rates were colistin (0%), ceftolozane/tazobactam (0%), and amikacin (42.5%). Given the time frame of the study and its retrospective nature, ceftolozane/tazobactam was only tested in six episodes. All ESBL-*K. pneumoniae* isolates were susceptible to imipenem and meropenem; three isolates were categorized as intermediate to ertapenem. Fifty-nine isolates (59%) were susceptible to piperacillin-tazobactam, eighteen (18%) to amoxicillin-clavulanic acid, thirteen (13%) to trimethoprim-sulfamethoxazole, and eight (8%) to ciprofloxacin. 

### 3.1. Primary Outcome: Clinical Failure

Clinical failure on day 7 of treatment was 28.7% (29/101) and 8% (8/100) in the XDR *P. aeruginosa* and ESBL-*K. pneumoniae* groups, respectively (*p* < 0.001). Failure in the XDR *P. aeruginosa* group was due to the persistence or worsening of signs and/or symptoms (twenty-six episodes), death (two episodes), and one episode in which therapy had to be modified due to the side effects of antibiotics. Causes of failure in the ESBL-*K. pneumoniae* group were death (five episodes), persistence or worsening of signs and/or symptoms (two cases), and one case of treatment modification due to the side effects of antibiotics. 

Clinical failure at the end of treatment was 19.8% (20/101) in the XDR *P. aeruginosa* cohort vs. 8% (8/100) in the ESBL-*K. pneumoniae* group (*p* = 0.016). Reasons for failure in the XDR *P. aeruginosa* group were persistence or worsening of signs and/or symptoms (nine episodes), need to modify therapy due to antibiotic side effects (four cases), isolation of a new strain of XDR *P. aeruginosa* resistant to antibiotic treatment (three episodes) and death (four patients). In all cases, the reason for switching antibiotic treatment following antibiotic side effects was nephrotoxicity. The reasons for failure in the ESBL-*K. pneumoniae* group were death (seven patients) and one patient with persistent or worsening of signs and/or symptoms.

The unadjusted and adjusted analysis of variables associated with clinical failure on day 7 and at EOT are shown in Table 2 and Table 3, respectively. After adjusting for confounders in multivariate analysis, XDR *P. aeruginosa* cUTI was independently associated with clinical failure on day 7 of treatment (OR 4.34, 95% CI 1.71–11.04; p = 0.002) but not at the end of treatment (OR 2.31, 95% CI 0.83–6.44; p 0.108). Clinical failure at 7 days was also independently associated with quick SOFA (OR 1.97, 95% CI 1.11–3.51; *p* = 0.020). After adjusting for confounders, clinical failure at the end of treatment was independently associated with UTI classification (acute pyelonephritis) (OR 0.00, 95% CI −0.00), Charlson index (OR 1.20, 95% CI 1.01–1.43; *p* = 0.036) and quick SOFA (OR 2.19, 95% CI 1.15–4.18; *p* = 0.017), but not with XDR *P. aeruginosa* infection.

### 3.2. Secondary Outcomes: Mortality, Microbiological Assessment, and Economic Analysis

The 30-day mortality rate was 9.9% (10/101) among patients with XDR *P. aeruginosa* cUTI and 13% (13/100) in patients with ESBL-*K. pneumoniae* infections. Ninety-day mortality was 24.8% (25/101) in XDR-*P. aeruginosa* and 23% (23/100) in ESBL-*K. pneumoniae*. Univariate and multivariate analyses of parameters associated with 30-day and 90-day mortality are shown in Table 4 and Table 5, respectively. In the adjusted analysis, XDR *P. aeruginosa* was not associated with increased 30-day or 90-day mortality; 30-day mortality was independently associated with age (HR 1.06, 95% CI 1.01–1.11; *p* = 0.020), Charlson index (HR 1.2, 95% CI 1.03–1.4; *p* = 0.020) and inadequate source control of infection at the onset of bacteraemia (HR 3.36, 95% CI 1.15–9.83; *p* = 0.027). After adjusting for confounders, 90-day mortality was independently associated with age (HR 1.03, 95% CI 1.00–1.07; *p* = 0.049), Charlson index (HR 1.38, 95%CI, 1.22–1.5; *p* = 0.00), and quick SOFA (HR 3.26, 95% CI 2.09–5.10; *p* = 0.000).

In terms of the microbiological assessment, eradication rates in patients with follow-up urine cultures performed within 90 days were higher in XDR *P. aeruginosa* infection than in ESBL-*K. pneumoniae* (51/77 (66.2%) vs 23/61 (37.7%), *p* = 0.001). The recurrence of cUTI at 90 days was higher in ESBL-*K. pneumoniae* patients than in those with XDR *P. aeruginosa*: 40 (40%) vs 18 (17.8%) (*p* = 0.001), as was 90-day infection-related readmission: 21 (52.5%) vs 16 (26.7%) (*p* = 0.009), respectively.

In terms of cost estimation, it was found that XDR *P. aeruginosa* relative to ESBL-*K. pneumoniae* was not associated with higher costs (median difference, MD = 3612.06 EUR; 95%CI −2204.66 to 9428.78; *p* = 0.222). Factors associated with higher costs were bacteraemia, nosocomial acquisition of infection, together with length of hospital stay, see Table 6. After multivariate analysis was performed, excluding length of hospital stay data to prevent the influence of this factor on cost estimation, the only two variables associated with higher costs were bacteraemia (MD = 16228.95 EUR; 95% CI 10394.83–22063.06; *p* < 0.001) and nosocomial acquisition (MD = 13401.76 EUR; 95%CI 8228.21–18575.31; *p* < 0.001).

### 3.3. Adverse Events

Acute renal failure occurred only in the XDR *P. aeruginosa* infection group, in 27 patients (26.7%) who received treatment with colistin (*n* = 17), amikacin (*n* = 4) and amikacin + colistin (*n* = 6). The incidence of *Clostridioides difficile* infection was similar in both groups: 4% in XDR *P. aeruginosa* versus 5% in the ESBL-*K. pneumoniae* group. Neurological complications were seen only in two patients in the ESBL-*K. pneumoniae* group: one patient, on ertapenem treatment, had seizures; and one patient, on imipenem treatment, had an altered level of consciousness and myoclonus.

## 4. Discussion

The objective of our study was to compare clinical characteristics, outcomes and economic differences in cUTI caused by XDR *P. aeruginosa* and ESBL-*K. pneumoniae*. We found that patients with XDR *P. aeruginosa* cUTI had worse early clinical cures and more antibiotic side effects. However, we found no differences in clinical failure at end of treatment, mortality, or economic costs between the two groups. ESBL-*K. pneumoniae* cUTIs were associated with worse eradication rates and higher 90-day infection-related readmission. Although previous studies have associated healthcare-related UTI with increased resource consumption [37] and worse outcomes [16], few have compared the clinical and economic burden of infections caused by XDR *P. aeruginosa* versus other MDR-GNB pathogens [23,24].

In the present study, urinary tract infections caused by ESBL-*K. pneumoniae* were observed more frequently in patients with diabetes mellitus, in kidney transplant recipients, and with co-existing renal failure. In a review of the literature, different authors have shown comparable results [38,39,40]. In two retrospective case-control studies conducted to identify risk factors for ESBL-*K. pneumoniae* UTI, Espinar *et al.* found that diabetes mellitus (*p* < 0.007) and patients with delayed graft function (*p* = 0.001) were independent risk factors in non-hospitalized kidney transplant patients [38], while Lautenbach *et al.* found a borderline significant association between ESBL-Enterobacterales infection and diabetes (*p* = 0.07). In another case-control study comparing non-ESBL-*K.pneumoniae* vs ESBL-*K.pneumoniae* UTIs, chronic kidney disease was found to be more prevalent in the second group (*p* = 0.0296) [39]. The risk factors for XDR *P. aeruginosa* infection in our study were COPD and solid tumour malignancy, two conditions which are already known to be associated with *P. aeruginosa* infections. As an opportunistic pathogen, *P. aeruginosa* usually infects patients with immunodeficiency, malignancy, or chronic pulmonary disorder. In patients with chronic inflammatory airway diseases, such as COPD, cystic fibrosis, asthma, or bronchiectasis, *P. aeruginosa* produces chronic colonization of the lower respiratory tract and respiratory infections [41]. In these patients, *P. aeruginosa* frequently colonizes the gastrointestinal tract as well [42,43], making them more susceptible to cUTI by these pathogens than by other bacterium. Regarding patients with solid malignancy, their immunocompromised status and the use of immunosuppressive therapy promote *P. aeruginosa* infection in these patients [44].

With respect to clinical outcomes, our study suggests that XDR *P. aeruginosa* cUTIs have worse early clinical cure rates than ESBL-*K. pneumoniae* infections. It is also worth pointing out that a significant number of treatment failures in this group were due to antibiotic nephrotoxicity. It is known that *P. aeruginosa* can develop drug resistance during prolonged therapy as early as three days after the initiation of therapy with an antibiotic to which it is originally tested as sensitive, which would also further explain the difficulty of treatment and/or the poorer clinical outcome associated with this organism. [45]. These results, in agreement with our hypothesis, support the statement that infections caused by XDR *P. aeruginosa* are always a difficult-to-treat scenario with very limited treatment options that lead to worse clinical outcomes.

In terms of mortality, after adjusting for cofounders, we were unable to find differences between the two groups. Mortality rates for XDR *P. aeruginosa* infection (30-day mortality 9.9%, 90-day mortality, 24.8%) were lower than in previous reports: 33.6% for 30-day mortality in XDR *P. aeruginosa* infections [46] and around 30% for MDR/XDR *P. aeruginosa* episodes [20,22,47], although these studies included bacteraemic episodes and sources of infection other than the urinary tract. The differences in mortality therefore may be explained by the lower risk foci of infection of urinary tract infection, and low mortality rates [48]. On the other hand, 30-day mortality in our study in the ESBL*-K. pneumoniae* group (13%) was similar to other studies of mortality in UTIs caused by this microorganism [25,40]. In a cohort study by Richelsen *et al.* [25] evaluating the impact of ESBL production on community-onset infections due to *Escherichia coli* or *K. pneumoniae*, 30-day mortality was 13.8%. In another study by Larisa-Miftode *et al.* [40] comparing ESBL and non- ESBL-K. *pneumoniae* infections, mortality in the ESBL group was 17.6%.

Many different studies on ESBL-Enterobacterales and *P. aeruginosa* bloodstream infections have found that time to appropriate antibiotic treatment is an independent predictor of mortality [49,50,51,52]. However, for complicated urinary tract infections, there is no clear evidence. In our study, we found a considerable difference between the two groups in time to perform appropriate therapy (72 h delay: 53.5% in XDR *P. aeruginosa* and 39% in ESBL-*K. pneumoniae*). These differences were expected because there are fewer available treatment alternatives against XDR *P. aeruginosa*. Nevertheless, we did not find a statistically significant association with clinical failure or mortality. One explanation could be the lack of severity in the initial presentation of infection in most patients; the frequency of sepsis or septic shock was 31.7% and 32.7%, respectively. Some authors also argue that, in cases of *P. aeruginosa* infection, a 48–72 h delay of receipt of the appropriate antibiotics does not have a great impact on patient outcome, since mortality is mainly due to other factors, such as clinical presentation, source of infection or receipt of inappropriate definitive antibiotic therapy [53,54,55].

In recent decades, the increase in multi-drug resistance has an impact not only on clinical outcomes but also on economic costs. It is estimated that the cost of infections caused by antimicrobial-resistant organisms is higher, ranging from $6000-$30,000, than in infections caused by antimicrobial-susceptible organisms [56]. While previous studies have compared resource consumption between susceptible and resistant microorganisms, we set out to compare economic differences between two multidrug-resistant bacteria. In a review of the literature, several reports have associated XDR *P. aeruginosa* infection with higher resource consumption [57], although in our economic analysis we found no differences between the two microorganisms. Our results could be partly explained by the worse eradication and recurrence rates observed in the ESBL*-K. pneumoniae* group and thus, higher hospital readmission rates. While *K. pneumoniae* is a normal part of the intestinal flora in humans, hospital patients are more susceptible to colonization by this bacterium, which further complicates eradication [58]. Another possible explanation is that the patients with the highest prevalence of ESBL-*K.pneumoniae* cUTI were kidney transplant recipients and those with chronic renal failure. Both these comorbidities have been identified in previous studies as risk factors associated with UTI recurrence [59]. We suggest that some of the strategies to prevent cUTI recurrence in these patients should be based on the incorporation of antimicrobial stewardship programmes in healthcare facilities, promoting catheter restriction protocols to limit catheter use and appropriate discontinuation of catheters [60].

In the economic study, after adjustment for cofounders, bacteraemia and nosocomial acquisition were found to be associated with higher costs, which is explained by the greater severity of the infection and longer length of stay, respectively.

Our study has the inherent limitations of a retrospective, single-center study in a specific health system, which may influence the applicability of the results. In addition, our results may not be generalizable to other geographical areas with different epidemiology, especially regarding differences in the most prevalent high-risk clones of both XDR *P. aeruginosa* and ESBL-*K. pneumoniae*. Second, due to the retrospective nature of the study, we did not conduct molecular clonality studies of the strains, which would have been useful to confirm the presence of certain high-risk clones in our cohort, and to study resistance determinants. Third, since carbapenemase-producing *K.pneumoniae* may share more similarities than ESBL with *XDR P. aeruginosa* in terms of a spectrum of resistance, clonal widespread, and nosocomial/HCA acquisition, it could be of interest to explore the role of carbapenemase-producing *K.pneumoniae* as comparator group. However, since the prevalence of ESBL-*K. pneumoniae* is much higher in our geographical area [61,62], ESBL may reflect more accurately the burden of MDR Enterobacteriae in the study setting.

Four, our study was conducted at a time when the “new” antibiotics against MDR-GNB ceftolozane/tazobactam and ceftazidime/avibactam were not routinely tested. Furthermore, as mentioned before, mortality rates in urinary tract infections tend to be low compared with other sources of infection and the results are not therefore generalizable to other sources of infection. Finally, antibiotic dosing was not recorded. This information would have been useful to evaluate the role of appropriate antibiotic exposures and the impact on adverse effects.

As a strength, while several previous reports have analysed the clinical differences between resistant and susceptible *P. aeruginosa* infections, to our knowledge, this is the first report to compare the clinical impact of XDR *P. aeruginosa* with another difficult-to-treat bacteria (ESBL-*K. pneumoniae)*, while also exploring the dimension of resource consumption.

## 5. Conclusions

Management of XDR *P. aeruginosa* infection is always challenging due to its intrinsic particularities and the limited treatment options available. In our study patients with XDR *P. aeruginosa* cUTI had worse early clinical cures and more antibiotic side effects, compared with ESBL-*K.pneumoniae.* We also found differences between the two groups in terms of appropriate time for empirical therapy, but there was no statistical association with clinical failure at the end of treatment or with mortality, probably explained by the low-risk foci of urinary tract infections. However, ESBL-*K. pneumoniae* cUTIs were associated with worse eradication rates, higher recurrence, and 90-day infection-related readmission. We did not find differences in either a clinical failure at the end of treatment, mortality, or economic costs between the two groups.

## Figures and Tables

**Table 1:** Baseline characteristics of patients included in the study and comparative analysis according to microorganism.

	Overall Cohort (*n* = 201)		
	XDR *P. aeruginosa* (*n* = 101)	ESBL-*K. pneumoniae* (*n* = 100)	*p*-Value
**Demographics**			
Age (years), m (IQR)	76 (67–82)	77 (64–83)	0.312
Male sex	80 (79.2)	65 (65)	0.025
**Underlying condition**			
Charlson comorbidity index, m (IQR)	7 (5–9)	6 (4.25–8)	0.150
Diabetes mellitus	30 (29.7)	46 (46)	0.017
COPD	31 (30.7)	16 (16)	0.014
Congestive heart failure	17 (16.8)	20 (20)	0.562
Cirrhosis	4 (4.0)	7 (7)	0.343
Neurological disorder	25 (24.8)	22 (22)	0.645
Hematologic malignancy	17 (16.8)	9 (9)	0.098
Solid tumor malignancy	49 (48.5)	24 (24)	0.000
**Immunosuppression**			
Neutropenia	6 (5.9)	5 (5)	0.769
**Nephro-urological history**			
Chronic kidney disease	25 (24.8)	40 (40)	0.021
Dialysis	6 (5.9)	1 (1)	0.118
Renal transplant	5 (5.0)	19 (19)	0.002
Benign prostatic hypertrophy	30 (29.7)	27 (27)	0.671
Obstructive urinary disease	12 (11.9)	14 (14)	0.654
Recurrent UTI	49 (48.5)	58 (58)	0.178
Indwelling urinary catheter in last 14 days	69 (68.3)	42 (42)	0.000
Other urological devices in last 14 days	18 (17.8)	17 (17)	0.878
Urological neoplasia	21 (20.8)	14 (14)	0.204
**UTI classification**			0.021
Acute pyelonephritis	8 (7.9)	19 (19)	
Complicated UTI	93 (92.1)	81 (81)	
Acute prostatitis	3 (3.2)	18 (22.5)	0.000
UTI due to indwelling urethral catheter	42 (44.2)	28 (35)	0.215
Obstructive uropathy	2 (2.1)	11 (13.8)	0.003
Neurogenic bladder	3 (3.2)	0 (0.0)	0.251
Urinary tract abnormalities	32 (33.7)	3 (3.8)	0.000
**Acquisition**			0.000
Community-acquired	0 (0.0)	21 (2)	0.000
Healthcare-related	51 (50.5)	49 (49)	0.832
Nosocomial	50 (49.5)	30 (30)	0.005
**HCA risk factors**			
Hospital stay in last 3 months	57 (56.4)	69 (69)	0.066
Surgery in last 3 months	38 (37.6)	24 (24)	0.037
ICU admission in last 3 months	22 (21.8)	8 (8)	0.006
Residence in long-term care facility	14 (13.9)	9 (9)	0.279
Antibiotic exposure in last 3 months	87 (86.1)	80 (80)	0.246
**Baseline illness severity**			
SOFA score, m (IQR)	1 (0–3)	2 (0–3)	0.260
qSOFA score, m (IQR)	0 (0–1)	0 (0–0.75)	0.374
SAPS II, m (IQR)	36 (28.5–41)	35.5 (28–42)	0.801
Sepsis or septic shock	32 (31.7)	33 (32.7)	0.880
ICU admission	12 (11.9)	9 (9)	0.504
Bacteremia	21 (20.8)	29 (29)	0.178
Pitt score, m (IQR)	1 (0–2)	0 (0–2)	0.969
**Management**			
Appropriate treatment—empirical	80 (79.2)	44 (45)	0.000
Appropriate treatment—definitive	97 (96.0)	98 (98.0)	0.683
72h delay to initiate appropriate antibiotic therapy	54 (53.5)	39 (39)	0.040
Inadequate source control	11 (10.9)	11 (11)	0.980
**Length of hospital stay (days) from onset of UTI, m (IQR)**	16 (11–22.5)	14 (8–21.75)	0.293

Data are presented as nos. (%), unless otherwise specified. Abbreviations: XDR (Extensively drug-resistant), ESBL (Extended-spectrum beta-lactamase-producing), COPD (chronic obstructive pulmonary disease), GFR (glomerular filtration rate), UTI (urinary tract infection), HCA (healthcare-acquired), ICU (intensive care unit), SAPS II (Simplified Acute Physiology Score), SOFA (Sequential Organ Failure Assessment), m (median), IQR (interquartile range).

**Table 2:** Univariate and multivariate analysis of parameters predicting clinical failure at day 7 of treatment.

	Overall Cohort (*n* = 201, Clinical Failure at Day 7-of Treatment = 37)					
	Clinical Failure (*n* = 37)	Non-Clinical Failure (*n* = 165)	Unadjusted OR (95% CI)	*p*-Value	Adjusted OR (95% CI)	*p*-Value
**Microorganism**						
XDR *P. aeruginosa*	29 (78.4)	72 (43.9)	4.63 (2.00–10.74)	<0.001	4.34 (1.71–11.04)	0.002
ESBL *K. pneumoniae*	8 (21.6)	92 (56.1)	0.22 (0.09–0.50)	<0.001		
**Demographic information**						
Age (years), m (IQR)	78 (71–85)	75 (65–82)	1.03 (0.99–1.07)	0.061	1.03 (0.99–1.07)	0.104
Male sex	28 (75.7)	117 (71.3)	1.25 (0.55–2.85)	0.596	0.99 (0.99–0.39)	0.999
**Underlying condition**						
Charlson comorbidity index, m(IQR)	8 (5–10)	6.50 (5–8.85)	1.09 (0.95–1.25)	0.189	1.07 (0.92–1.25)	0.373
Diabetes Mellitus	10 (27)	66 (40.2)	0.55 (0.25–1.21)	0.138		
COPD	8 (21.6)	39 (23.8)	0.88 (0.37–2.09)	0.779		
Congestive heart failure	8 (21.6)	29 (17.7)	1.28 (0.53–3.09)	0.577		
Cirrhosis	3 (8.1)	8 (4.9)	1.72 (0.43–6.82)	0.440		
Neurological disorder	7 (18.9)	40 (24.4)	0.72 (0.30–1.77)	0.479		
Hematologic malignancy	10 (27.0)	16 (9.8)	3.42 (1.40–8.34)	0.007		
Solid tumor malignancy	17 (45.9)	56 (34.1)	1.63 (0.80–3.37)	0.180		
**Immunosuppression**						
Neutropenia	2 (5.4)	9 (5.5)	0.98 (0.20–4.75)	0.984		
**Nephro-urological history**						
Chronic kidney disease	14 (37.8)	51 (31.1)	1.34 (0.64–2.83)	0.430		
Dialysis	5 (13.5)	2 (1.2)	12.65(2.35–68.11)	0.003		
Renal transplant	1 (2.7)	23 (14.0)	0.17 (0.22–1.30)	0.088		
Benign prostatic hypertrophy	12 (32.4)	45 (27.4)	1.26 (0.58–2.73)	0.543		
Obstructive urinary disease	6 (16.2)	20 (12.2)	1.39 (0.51–3.75)	0.512		
Recurrent UTI	19 (51.4)	88 (53.7)	0.91 (0.44–1.86)	0.799		
Indwelling urinary catheter in last 14 days	21 (56.8)	90 (54.9)	1.08 (0.52–2.21)	0.836		
Other urological devices in last 14 days	6 (16.2)	29 (17.7)	0.90 (0.34–2.35)	0.832		
Urological neoplasia	8 (21.6)	27 (16.5)	1.40 (0.57–3.40)	0.456		
**UTI classification**						
Acute pyelonephritis	3 (8.1)	24 (14.6)	0.51 (0.14–1.81)	0.301		
Complicated UTI	35 (92.1)	139 (85.3)	1.94 (0.55–6.83)	0.301		
Acute prostatitis	3 (8.6)	18 (12.9)	0.63 (0.17–2.29)	0.488		
UTI due to indwelling urethral catheter	15 (42.9)	55 (39.3)	1.15 (0.54–2.45)	0.700		
Obstructive uropathy	4 (11.4)	9 (6.4)	1.87 (0.54–6.50)	0.320		
Neurogenic bladder	0 (0)	3 (2.1)	-	-		
Urinary tract abnormalities	6 (17.1)	29 (20.7)	0.79 (0.30–2.08)	0.637		
**Acquisition**						
Community-acquired	3 (8.1)	18 (11)	1.05 (0.27–4.04)	0.933		
Healthcare-related	15 (40.5)	85 (51.8)	1.06 (0.27–4.04)	0.933		
Nosocomial	19 (51.4)	61 (37.2)	0.56 (0.27–1.15)	0.115		
**HCA risk factors**						
Hospital stay in last 3 months	19 (51.4)	107 (65.2)	0.56 (0.27–1.15)	0.117		
Surgery in last 3 months	8 (21.6)	54 (32.9)	0.56 (0.24–1.31)	0.183		
ICU admission in last 3 months	5 (13.5)	25 (15.2)	0.87 (0.30–2.44)	0.790		
Residence in long-term care	4 (10.8)	19 (11.6)	0.92 (0.30–2.90)	0.894		
Antibiotic exposure in last 3 months	31 (83.8)	136 (82.9)	1.06 (0.40–2.80)	0.900		
**Baseline illness severity**						
SOFA score, m (IQR)	2 (1–4)	1 (0.00–3)	1.06 (0.95–1.20)	0.303		
qSOFA score, m (IQR)	0.00 (0.00–1)	0.00 (0.00–1)	2.08 (1.22–3.55)	0.007	1.97 (1.11–3.51)	0.020
SAPS II	36 (29.50–45)	36 (28–41)	1.03 (1.00–1.07)	0.022		
Sepsis or septic shock	15 (40.5)	50 (30.5)	1.55 (0.74–3.24)	0.240		
ICU admission	4 (10.8)	17 (10.4)	1.04 (0.33–3.32)	0.936		
Bacteremia	11 (29.7)	39 (23.8)	1.35 (0.61–2.99)	0.451		
Pitt score, m (IQR)	1 (1–4)	0 (0–2.00)	1.62 (1.05–2.50)	0.027		
**Management**						
Appropriate empirical treatment	9 (24.3)	67 (40.9)	0.46 (0.20–1.05)	0.065	1.07 (0.33–3.48)	0.906
Appropriate definitive treatment	35 (94.6)	160 (97.6)	0.43 (0.77–2.48)	0.351		
72h delay to initiate appropriate antibiotic treatment	21 (56.8)	72 (43.9)	1.67 (0.81–3.44)	0.159	1.36 (0.48–3.83)	0.549
Inadequate source control	6 (16)	16 (9.8)	1.79 (0.65–4.94)	0.261	1.86 (0.59–5.87)	0.288

Data are presented as nos. (%), unless otherwise specified. Abbreviations: XDR (Extensively drug-resistant), ESBL (Extended-spectrum beta-lactamase-producing), COPD (chronic obstructive pulmonary disease), GFR (glomerular filtration rate), UTI (urinary tract infection), HCA (healthcare-acquired), ICU (intensive care unit), SAPS II (Simplified Acute Physiology Score), SOFA (Sequential Organ Failure Assessment), m (median), IQR (interquartile range).

**Table 3:** Univariate and multivariate analysis of parameters predicting clinical failure at end-of treatment.

	Overall Cohort (*n* = 201, Clinical Failure at End of Treatment = 28)					
	Clinical Failure (*n* = 28)	Non-Clinical Failure (*n* = 173)	Unadjusted OR (95% CI)	*p*-Value	Adjusted OR (95% CI)	*p*-Value
**Microorganism**						
XDR *P. aeruginosa*	20 (71.4)	81 (46.8)	2.84 (1.18–6.79)	0.019	2.31 (0.83–6.44)	0.108
ESBL-*K. pneumoniae*	8 (28.6)	92 (53.2)	0.35 (0.14–0.84)	0.019		
**Demographic information**						
Age (years), m (IQR)	76 (69–87)	76 (65–82)	1.03 (0.99–1.07)	0.103	1.03 (0.99–1.08)	0.134
Male sex	20 (71.4)	125 (72.3%)	0.96 (0.39–2.32)	0.928	0.71 (0.26–1.96)	0.515
**Underlying condition**						
Charlson comorbidity index, m (IQR)	8 (7–9.75)	6 (5–8)	1.20 (1.03–1.39)	0.017	1.20 (1.01–1.43)	0.036
Diabetes Mellitus	9 (32.1)	67 (38.7)	0.75 (0.32–1.75)	0.506		
COPD	6 (21.4)	41 (23.7)	0.87 (0.33–2.31)	0.792		
Congestive heart failure	9 (32.1)	28 (16.2)	2.45 (1.00–5.97)	0.048		
Cirrhosis	3 (10.7)	8 (4.6)	2.47 (0.61–9.95)	0.202		
Neurological disorder	9 (32.1)	38 (22)	1.68 (0.70–4.02)	0.241		
Hematologic malignancy	5 (17.9)	21 (12.1)	1.57 (0.54–4.58)	0.406		
Solid tumor malignancy	15 (53.6)	58 (33.5)	2.28 (1.02–5.12)	0.044		
**Immunosuppression**						
Neutropenia	1 (3.6)	10 (5.8)	0.60 (0.74–4.90)	0.637		
**Nephro-urological history**						
Chronic kidney disease	11 (39.3)	54 (31.2)	1.42 (0.62–3.25)	0.399		
Dialysis	2 (7.1)	5 (2.9)	2.58 (0.47–14.02)	0.271		
Renal transplant	0 (0)	24 (13.9)	-	-		
Benign prostatic hypertrophy	8 (28.6)	49 (28.3)	1.01 (0.41–2.45)	0.978		
Obstructive urinary disease	5 (17.9)	21 (12.1)	1.57 (0.54–4.58)	0.406		
Recurrent UTI	17 (60.7)	90 (52)	1.42 (0.63–3.22)	0.394		
Indwelling urinary catheter in last 14 days	16 (57.1)	95 (54.9)	1.09 (0.48–2.45)	0.826		
Other urological devices in last 14 days	5 (17.9)	30 (17.3)	1.03 (0.36–2.94)	0.947		
Urological neoplasia	8 (28.6)	27 (15.6)	2.16 (0.86–5.41)	0.099		
**UTI classification**						
Acute pyelonephritis	0 (0)	27 (15.6)	-	-		
Complicated UTI	28 (100)	146 (84.4)	-	-		
Acute prostatitis	2 (7.1)	19 (12.9)	0.51 (0.11–2.36)	0.396		
UTI due to indwelling urethral catheter	11 (39.3)	59 (40.1)	0.96 (0.42–2.20)	0.933		
Obstructive uropathy	2 (7.1)	11 (7.5)	0.95 (0.20–4.54)	0.950		
Neurogenic bladder	0 (0)	3 (2)	-	-		
Urinary tract abnormalities	7 (25)	28 (19)	1.41 (0.54–3.66)	0.472		
**Acquisition**						
Community-acquired	2 (7.1)	19 (11)	0.70 (0.14–3.38)	0.662		
Healthcare-related	13 (46.4)	87 (50.3)	1.42 (0.30–6.81)	0.662		
Nosocomial	13 (46.4)	67 (38.7)	1.37 (0.61–3.06)	0.441		
**HCA risk factors**						
Hospital stay in last 3 months	14 (50)	112 (64.7)	0.54 (0.24–1.21)	0.138		
Surgery in last 3 months	3 (10.7)	59 (34.1)	0.23 (0.06–0.80)	0.021		
ICU admission in last 3 months	4 (14.3)	26 (15)	1.52 (0.47–4.93)	0.477		
Residence in long-term care	4 (14.3)	19 (11)	1.35 (0.42–4.31)	0.612		
Antibiotic exposure in last 3 months	25 (89.3)	142 (82.1)	1.81 (0.51–6.40)	0.352		
**Baseline illness severity**						
SOFA score, m (IQR)	2 (1–3)	1 (0–3)	1.06 (0.93–1.20)	0.330		
q SOFA score, m (IQR)	1 (0.00–1)	0.00 (0.00–1)	2.24 (1.26–3.96)	0.006	2.19 (1.15–4.18)	0.017
SAPS II	36.50 (32–45.75)	36 (28–41)	1.03 (1.00–1.07)	0.033		
Sepsis or septic shock	11 (39.3)	54 (31.2)	1.42 (0.62–3.25)	0.399		
ICU admission	4 (14.3)	17 (9.8)	1.52 (0.47–4.93)	0.477		
Bacteremia	9 (32.1)	41 (23.7)	1.52 (0.64–3.62)	0.340		
Pitt score, m (IQR)	1 (1–4)	0.00 (0.00–2.00)	1.58 (1.02–2.44)	0.041		
**Management**						
Appropriate empirical treatment	6 (21.4)	70 (40.5)	0.40 (0.15–1.04)	0.060	1.43 (0.33–6.21)	0.635
Appropriate definitive treatment	28 (100)	167 (96.5)	-	-		
72h delay starting appropriate antibiotic treatment	17 (60.7)	76 (43.9)	1.97 (0.87–4.45)	0.103	1.43 (0.40–5.07)	0.575
Inadequate source control	6 (21.4)	16 (9.2)	2.67 (0.94–7.56)	0.063	2.38 (0.68–8.29)	0.174

Data are presented as nos. (%), unless otherwise specified. Abbreviations: XDR (Extensively drug-resistant), ESBL (Extended-spectrum beta-lactamase-producing), COPD (chronic obstructive pulmonary disease), GFR (glomerular filtration rate), UTI (urinary tract infection), HCA (healthcare-acquired), ICU (intensive care unit), SAPS II (Simplified Acute Physiology Score), SOFA (Sequential Organ Failure Assessment), m (median), IQR (interquartile range).

**Table 4:** Univariate and multivariate analysis of parameters predicting 30-day mortality.

	Overall Cohort (*n* = 201, 30-Day Mortality = 23)					
	Deaths (*n* = 23)	Alive (*n*= 178)	Unadjusted OR (95% CI)	*p*-Value	Adjusted OR (95% CI)	*p*-Value
**Microorganism**						
XDR *P. aeruginosa*	10 (43.5)	91 (51.5)	0.73 (0.30–1.76)	0.491	0.73 (0.30–1.78)	0.487
ESBL-*K. pneumoniae*	13 (56.5)	87 (48.9)	1.36 (0.56–3.26)	0.491		
**Demographic information**						
Age (years), m (IQR)	79 (73–87)	75 (65–82)	1.05 (1.00–1.09)	0.041	1.06 (1.01–1.11)	0.020
Male sex	15 (65.2)	130 (73)	0.69 (0.27–1.73)	0.433	0.65 (0.26–1.56)	0.341
**Underlying condition**						
Charlson comorbidity index, m(IQR)	9 (7–10)	6.50 (5–8)	1.20 (1.02–1.41)	0.026	1.20 (1.03–1.40)	0.020
Diabetes Mellitus	7 (30.4)	69 (38.8)	0.69 (0.27–1.76)	0.440		
COPD	6 (26.1)	41 (23)	1.17 (0.43–3.18)	0.745		
Congestive heart failure	8 (34.8)	29 (16.3)	2.74 (1.06–7.05)	0.037		
Cirrhosis	2 (8.7)	9 (5.1)	1.78 (0.36–8.83)	0.476		
Neurological disorder	6 (26.1)	41 (23)	1.17 (0.43–3.18)	0.745		
Hematologic malignancy	3 (13)	23 (12.9)	1.01 (0.27–3.67)	0.987		
Solid tumor malignancy	12 (52.2)	61 (34.3)	2.09 (0.87–5.01)	0.098		
**Immunosuppression**						
Neutropenia	0 (0)	11 (6.2)	-	-		
**Nephro-urological history**						
Chronic kidney disease	9 (39.1)	56 (31.5)	1.40 (0.57–3.42)	0.461		
Dialysis	2 (8.7)	5 (2.8)	3.29 (0.60–18.06)	0.169		
Renal transplant	0 (0.0)	23 (13)	-	-		
Benign prostatic hypertrophy	8 (34.8)	49 (27.5)	1.40 (0.56–3.52)	0.469		
Obstructive urinary disease	3 (13)	23 (12.9)	1.01 (0.27–3.67)	0.987		
Recurrent UTI	14 (60.9)	93 (52.2)	1.42 (0.58–3.45)	0.437		
Indwelling urinary catheter in last 14 days	10 (43.5)	101 (56.7)	0.58 (0.24–1.40)	0.233		
Other urological devices in last 14 days	3 (13)	32 (18)	0.68 (0.19–2.44)	0.559		
Urological neoplasia	7 (30.4)	28 (15.7)	2.34 (0.88–6.21)	0.087		
**UTI classification**				0.325		
Acute pyelonephritis	1 (4.3)	26 (14.6)	0.26 (0.34–2.05)	0.204		
Complicated UTI	22 (95.7)	152 (84.9)	3.76 (0.48–29.13)	0.204		
Acute prostatitis	2 (9.5)	19 (12.3)	0.74 (0.16–3.46)	0.711		
UTI due to indwelling urethral catheter	6 (28.6)	64 (41.6)	0.56 (0.20–1.52)	0.259		
Obstructive uropathy	2 (9.5)	11 (7.1)	1.36 (0.28–6.64)	0.697		
Neurogenic bladder	0 (0)	3 (1.9)	-	-		
Urinary tract abnormalities	4 (19)	31 (20.1)	0.93 (0.29–2.97)	0.907		
**Acquisition**						
Community-acquired	3 (13)	18 (10.1)	1.34 (0.34–5.32)	0.670		
Healthcare-related	11 (47.8)	89 (50)	0.74 (0.18–2.92)	1.000		
Nosocomial	9 (39.1)	71 (39.7)	0.96 (0.39–2.35)	0.944		
**HCA risk factors**						
Hospital stay in last 3 months	16 (69.6)	110 (61.8)	1.41 (0.55–3.61)	0.469		
Surgery in last 3 months	4 (17.4)	58 (32.6)	0.43 (0.14–1.33)	0.147		
ICU admission in last 3 months	1 (4.3)	29 (16.3)	0.23 (0.30–1.80)	0.163		
Residence in long-term care	6 (26.1)	17 (9.5)	3.34 (1.16–9.61)	0.025		
Antibiotic exposure in last 3 months	21 (91.3)	146 (82)	2.30 (0.51–10.31)	0.276		
**Baseline illness severity**						
SOFA score, m (IQR)	3 (2–5)	1 (0–3)	1.11 (0.98–1.26)	0.082	1.10 (0.99–1.22)	0.064
q-SOFA score, m (IQR)	1 (0.00–1)	0.00 (0.00–1)	4.05 (2.10–7.81)	<0.001		
SAPS II	40 (33–53)	35 (28–41)	1.07 (1.03–1.11)	<0.001		
Sepsis or septic shock	13 (56.5)	52 (29.2)	2.73 (1.39–5.33)	0.003		
ICU admission	5 (21.7)	16 (9)	2.81 (0.92–8.58)	0.069		
Bacteremia	7 (30.4)	43 (24.3)	1.37 (0.53–3.55)	0.514		
Pitt score, m (IQR)	2 (1–5)	0.00 (0.00–2.00)	1.74 (1.08–2.81)	0.022		
**Management**						
Appropriate empirical treatment	7 (30.4)	69 (38.8)	0.69 (0.27–1.76)	0.440	0.94 (0.22–4.01)	0.937
Appropriate definitive treatment	23 (100)	172 (96.6)	-	-		
72h delay to initiate appropriate antibiotic treatment	14 (60.9)	79 (44.4)	1.94 (0.80–4.73)	0.141	2.25 (0.59–8.6)	0.236
Inadequate source control	5 (21.7)	17 (9.6)	3.09 (1.24–7.70)	0.015	3.36 (1.15–9.83)	0.027

Data are presented as nos. (%), unless otherwise specified. Abbreviations: XDR (Extensively drug-resistant), ESBL (Extended-spectrum beta-lactamase-producing), COPD (chronic obstructive pulmonary disease), GFR (glomerular filtration rate), UTI (urinary tract infection), HCA (healthcare-acquired), ICU (intensive care unit), SAPS II (Simplified Acute Physiology Score), SOFA (Sequential Organ Failure Assessment), m (median), IQR (interquartile range).

**Table 5:** Univariate and multivariate analysis of parameters predicting 90-day mortality.

	Overall Cohort (*n* = 201, 90-Day Mortality = 48)					
	Deaths (*n* = 48)	Alive (*n* = 153)	Unadjusted OR (95% CI)	*p*-Value	Adjusted OR (95% CI)	*p*-Value
**Microorganism**						
XDR *P. aeruginosa*	25 (52.1)	76 (49.7)	1.10 (0.57–2.10)	0.771	0.86 (0.45–1.64)	0.656
ESBL-*K. pneumoniae*	23 (47.9)	77 (50.3)	0.90 (0.47–1.73)	0.771		
**Demographic information**						
Age (years), m (IQR)	76 (65–83)	76 (66–83)	1.00 (0.98–1.03)	0.547	1.03 (1.00–1.07)	0.049
Male sex	33 (68.8)	112 (73.2)	0.80 (3.97–1.63)	0.549		
**Underlying condition**						
Charlson comorbidity index, m(IQR)	9 (6.25–10)	6 (4–8)	1.40 (1.21–1.62)	<0.001	1.38 (1.22–1.55)	0.000
Diabetes Mellitus	17 (35.4)	59 (38.6)	0.87 (0.44–1.71)	0.695		
COPD	14 (29.2)	33 (21.6)	1.49 (0.72–3.11)	0.280		
Congestive heart failure	10 (20.8)	27 (17.6)	1.22 (0.54–2.76)	0.620		
Cirrhosis	5 (10.4)	6 (3.9)	2.84 (0.82–9.79)	0.096		
Neurological disorder	11 (22.9)	36 (23.5)	0.96 (0.44–2.08)	0.930		
Hematologic malignancy	7 (14.6)	19 (12.4)	1.20 (0.47–3.06)	0.697		
Solid tumor malignancy	31 (64.6)	42 (27.5)	4.81 (2.41–9.60)	<0.001		
**Immunosupression**						
Neutropenia	5 (10.4)	6 (3.9)	2.84 (0.82–9.79)	0.096	2.59 (0.90–7.48)	0.079
**Nephro-urological history**						
Chronic kidney disease	15 (31.3)	50 (32.7)	0.93 (0.46–1.88)	0.853		
Dialysis	3 (6.3)	4 (2.6)	2.48 (0.53–11.51)	0.245		
Renal transplant	1 (2.1)	23 (15)	0.12 (0.16–0.91)	0.041		
Benign prostatic hypertrophy	13 (27.1)	44 (28.8)	0.92 (0.44–1.90)	0.822		
Obstructive urinary disease	8 (16.7)	18 (11.8)	1.50 (0.60–3.70)	0.380		
Recurrent UTI	24 (50)	83 (54.2)	0.84 (0.44–1.61)	0.607		
Indwelling urinary catheter in last 14 days	22 (45.8)	89 (58.2)	0.60 (0.31–1.16)	0.136		
Other urological devices in last 14 days	9 (18.8)	26 (17)	1.12 (0.48–2.60)	0.780		
Urological neoplasia	11 (22.9)	24 (15.7)	1.59 (0.71–3.56)	0.252		
**UTI classification**						
Acute pyelonephritis	5 (10.4)	22 (14.4)	0.69 (0.24–1.94)	0.484		
Complicated UTI	43 (89.6)	131 (85.6)	1.44 (0.51–4.04)	0.484		
Acute prostatitis	4 (9.5)	17 (12.8)	0.71 (0.22–2.26)	0.572		
UTI due to indwelling urethra catheter	12 (28.6)	58 (43.6)	0.51 (0.24–1.09)	0.086		
Obstructive uropathy	5 (11.9)	8 (6)	2.11 (0.65–6.84)	0.213		
Neurogenic bladder	1 (2.4)	2 (1.5)	1.59 (0.14–18.07)	0.705		
Urinary tract abnormalities	10 (23.8)	25 (18.8)	1.35 (0.58–3.10)	0.480		
**Acquisition**						
Community acquired	3 (6.3)	18 (11.8)	0.50 (0.14–1.77)	0.284		
Healthcare-related	25 (52.1)	75 (49)	1.13 (0.59–2.16)	0.711		
Nosocomial	20 (41.7)	60 (39.2)	1.10 (0.57–2.14)	0.762		
**HCA risk factors**						
Hospital stay in last 3 months	30 (62.5)	96 (62.5)	0.99 (0.50–1.93)	0.976		
Surgery in last 3 months	10 (20.8)	52 (34)	0.51 (0.23–1.10)	0.089		
ICU admission in last 3 months	6 (12.5)	24 (15.7)	0.76 (0.29–2.00)	0.590		
Residence in long-term care	9 (18.8)	14 (9.2)	2.29 (0.92–5.69)	0.074		
Antibiotic exposure in last 3 months	43 (89.6)	124 (81)	2.01 (0.73–5.52)	0.175		
**Baseline illness severity**						
SOFA score, m (IQR)	2 (1–5)	1 (0–3)	1.11 (0.99–1.25)	0.066		
q SOFA score, m (IQR)	1 (0.00–1)	0.00 (0.00–0.00)	3.09 (1.79–5.33)	<0.001	3.26 (2.09–5.10)	0.000
SAPS II	41 (33–48.75)	27 (34–40)	1.07 (1.03–1.10)	<0.001		
Sepsis or septic shock	24 (50)	41 (26.8)	2.73 (1.39–5.33)	0.003		
ICU admission	9 (18.8)	12 (7.8)	2.71 (1.06–6.9)	0.031		
Bacteremia	18 (37.5)	32 (20.9)	2.26 (1.1–4.57)	0.022	1.60 (0.83–3.10)	0.161
Pitt score, m (IQR)	1 (0,00–3)	0.00 (0.00–2.00)	1.14 (0.95–2.10)	0.083		
**Management**						
Appropriate empirical treatment	20 (41.7)	56 (36.6)	1.23 (0.63–2.39)	0.528	0.51 (0.20–1.33)	0.171
Appropriate definitive treatment	48 (100)	147 (96.1)	-	0.999		
72h delay to initiate appropriate antibiotic treatment	23 (47.9)	70 (45.8)	1.09 (0.57–2.08)	0.793	1.72 (0.70–4.26)	0.238
Inadequate source control	10 (20.8)	12 (7.8)	3.09 (1.24–7.7)	0.015	1.91 (0.90–4.07)	0.091

Data are presented as nos. (%), unless otherwise specified. Abbreviations: XDR (Extensively drug-resistant), ESBL (Extended-spectrum beta-lactamase-producing), COPD (chronic obstructive pulmonary disease), GFR (glomerular filtration rate), UTI (urinary tract infection), HCA (healthcare-acquired), ICU (intensive care unit), SAPS II (Simplified Acute Physiology Score), SOFA (Sequential Organ Failure Assessment), m (median), IQR (interquartile range).

**Table 6:** Univariate and multivariate analysis of the economic impact (total costs in euros) per hospital admission according to different variables.

Variables	Univariate MD (95% CI)	*p*-Value	Multivariate MD (95% CI)	*p*-Value
XDR *P. aeruginosa*	3612.06 (−2204.66, 9428.78)	0.222	3003.16 (−2216.81, 8223.13)	0.258
Bacteremia	16744.23 (10159.42, 23329.04)	<0.001	16228.95 (10394.83, 22063.06)	<0.001
Nosocomial acquisition	15521.47 (9229.19, 21813.75)	<0.001	13401.76 (8228.21, 18575.31)	<0.001
Appropriate empirical treatment	5023.74 (−994.78, 11042.26)	0.101		
Charlson comorbidity index	276.42 (−944.95, 1497.79)	0.656	100.64 (−912.31, 1113.60)	0.845
Age	−107.81 (−335.44, 119.83)	0.351	21.48 (−196.27, 239.24)	0.846
SOFA	881.76 (−153.23, 1916.75)	0.094	613.66 (−313.33, 1540.66)	0.193
Length of hospital stay (days) from onset of UTI	602.95 (544.58, 661.32)	<0.001		

Abbreviations: MD (Median Difference)*,* XDR (Extensively drug-resistant), SOFA (Sequential Organ Failure Assessment), UTI (urinary tract infection).

## Data Availability

The data presented in this study is available in the article.
